# Assessment of plant water status in winter wheat (*Triticum aestivum* L.) based on canopy spectral indices

**DOI:** 10.1371/journal.pone.0216890

**Published:** 2019-06-10

**Authors:** Hui Sun, Meichen Feng, Lujie Xiao, Wude Yang, Chao Wang, Xueqin Jia, Yu Zhao, Chunqi Zhao, Saleem Kubar Muhammad, Deying Li

**Affiliations:** 1 Institute of Dry Farming Engineering, Shanxi Agricultural University, Taigu, Shanxi, China; 2 Department of Plant Sciences, North Dakota State University, Fargo, North Dakota, United States of America; College of Agricultural Sciences, UNITED STATES

## Abstract

Rapid and non-destructive estimation of plant water status is essential for adjusting field practices and irrigation schemes of winter wheat. The objective of this study was to find new combination spectral indices based on canopy reflectance for the estimation of plant water status. Two experiments with different irrigation regimes were conducted in 2015–2016 and 2016–2017. The canopy spectra were collected at different growth stages of winter wheat. The raw and derivative reflectance of canopy spectra showed obvious responses to the change of plant water status. Except for equivalent water thickness (EWT), other water metrics had good relationships with new combination spectral indices (R^2^>0.7). An acceptable model of canopy water content (CWC) was established with the best spectral index (RVI (1605, 1712)). Models of leaf water content (LWC) and plant water content (PWC) had better performances. Optimal spectral index of LWC was FDRVI (687, 531), having R^2^, RMSE and RPD of 0.77, 2.181 and 2.09; R^2^, RMSE and RPD of 0.87, 2.652 and 2.34 for calibration and validation, respectively. And PWC could be well estimated with FDDVI (688, 532) (R^2^, RMSE and RPD of 0.79, 3.136 and 2.21; R^2^, RMSE and RPD of 0.83, 3.702 and 2.18 for calibration and validation, respectively). Comparing the performances of estimation models, the new combination spectral indices FDRVI (687, 531) based on canopy reflectance improved the accuracy of estimation of plant water status. Besides, based on FDRVI (687, 531), LWC was the optimal water metrics for plant water status estimation.

## Introduction

Drought interrupts agricultural production and other human activities at global as well as regional scales [1, 2]. Water shortage can happen during major drought events and in areas with scarce water supply. Increasing water efficiently in irrigation is one of the many strategies to deal with water shortage and conserve water. Real-time assessment of crop water status is essential for irrigation scheduling because plant water status is a sensitive indicator prior to changes in morphological and physiological responses to water stress [3, 4].

Remote sensing technology has been extensively used for non-destructive and quick assessment of crop growth condition as an alternative to more conventional means. Because water status of plants can directly influence cell turgor, chemical reactions, and the arrangement of cell and tissues in leaves, the absorption, transmission, and reflection of light from an individual leaf and leaf canopy are also affected by plant water status. For example, the light reflectance of a cotton (*Gossypium hirsutum* L.) canopy was used to predict the physiological status of cotton plants under water stress by Jackson and Ezra [5]. Attempt has been made in recent years to use the technology for fast detection of changes of water status in field plants [6–8]. Quantifying plant water status using remote sensing requires model building and ground testing. Peñuelas et al. [8, 9] used plant water content and the leaf relative water content to test the model developed from spectral reflectance data. At a canopy level and in larger scales, the quantification is more often expressed as equivalent water thickness (EWT) and canopy water content (CWC) [10, 11].

The primary factors influencing the reliability of water prediction models based on hyperspectral data are model statistical approaches as well as spectrum and the spectral characteristic parameters. The multiple regression models or machine learning models with either full reflective spectrum or sensitive wavelengths could adequately estimate the water status of plants [12–14]. The absorption features (e.g. peak position and height) of water absorption bands also showed good correlation with the relative water content of different plants [15–17]. Based on those features, different vegetation indices were proposed to enhance the vegetation information while minimize the influences from solar irradiance changes or soil brightness [18]. For instance, Hunt and Rock [19] found a good correlation between EWT and the moisture stress index (R_1600_/R_820_). Other indices include normalized different water index (NDWI) (R_860_-R_1240_)/(R_860_+R_1240_) by Gao [20], water index (WI) (R_900_/R_970_) by Panigada, Rossini [21], water index (normalized different index and ratio index calculated with the reflectance at 780 nm and 1750 nm) [22], and more [23].

Other factors affecting the plant water prediction models are canopy structure and soil background. The soil influences are particularly significant when with the lack of complete vegetation coverage, such as during the early stages of crop growth and when the crops are under stress. Huete [24] proposed the soil-adjusted vegetation index (SAVI) to minimize the effect of the soil surface. Subsequently, Rondeaux and Steven [25] modified the parameter *L* in the SAVI and arrived at an optimal soil-adjusted vegetation index (OSAVI). Most recently, derivative transformation of the spectrum data are used to suppress the spectral response to soil background [26].

The objective of this study was to evaluate different models in predicting the water status of winter wheat based on canopy reflectance with or without derivative transformation. Ultimately, our goal was to select an optimal water metrics and spectral index in assessing the water status of winter wheat.

## Materials and methods

### Site description and experimental design

The experiment was conducted from 2015 to 2017 at the experiment station of Shanxi Agricultural University (E112°33’, N37°25’), Shanxi Province, China. The experiment site has a temperate continental climate with an average annual temperature of 9.8 °C, 175 frost-free days and annual precipitation about 450 mm.

There were two winter wheat cultivars (Chang 4738 and Zhongmai 175) during the 2015–2016 growing season. Only one cultivar (Jingdong 17) was included during the 2016–2017 growing season. Wheat seeds were sown at a density of 400 plant m^-2^ on September 29 and October 1 in 2015 and 2016, respectively. The experiments were carried out in a bottomless water-experiment pool (10 m×9 m) buried in the ground. The refilled soil in the pool was classified as Calcareous Cinnamon soil (Alfisols in US taxonomy) with 9.60 g kg^-1^ organic matters, 57.75 g kg^-1^ available nitrogen, 22.10 mg kg^-1^ available phosphate, and 185.48 mg kg^-1^ available potassium. The field capacity of this artificial root zone was 24.24% at the bulk density of 1.42 g cm^-3^.

There were five irrigation regimes included in the two-year experiment: I_1_ (four irrigations at jointing stage, booting stage, flowering stage, and filling stage), I_2_ (three irrigations at jointing stage, booting stage, and filling stage), I_3_ (two irrigations at jointing stage and flowering stage), I_4_ (two irrigations at jointing stage and booting stage), and I_5_ (without irrigation). The amount of water at each irrigation was 80% of the soil field capacity controlled with a water meter. The growth stages for irrigation were selected based on previous studies [27]. For all treatments, the fertilizers were applied prior to seeding with 150 kg N hm^-2^, 150 kg P_2_O_5_ hm^-2^, and 150 kg K_2_O hm^-2^. The experiment was set up in a randomized complete block design with three replications.

### Canopy reflectance measurement

The canopy spectral reflectance was collected from jointing stage to filling stage using a FieldSpec 3.0 Spectrometer (Analytical Spectral Devices (ASD), Boulder, CO, USA) at 1 m above the canopy. The spectral range collected was 350–2500 nm, with a sampling interval of 1.4 nm and spectral resolution of 3 nm between 350 and 1000 nm; and a sampling interval of 2 nm and spectral resolution of 10 nm between 1000 and 2500 nm. The measurements were taken under clear sky conditions from 10:00 to 14:00 hours. Three measurements were taken in each plot. Ten reflectance curves per site were averaged. A 40 cm square BaSO_4_ panel was used for calibrating the baseline reflectance before each measurement.

### Plant water status measurement

Plant samples were collected at the same time of spectral measurements. Plants from 400 cm^2^ area in each plot were clipped at the soil surface and stored in plastic bags before the measurement of water content. The fresh plant was separated into different organs (leaf, stem and spike) and weighed for fresh weight (FW). Samples were dried in an oven at 105 °C for half an hour, then at 80 °C for 24 hours to constant weight and reweighed to get dry weight (DW). Leaf water content (LWC) and Plant water content (PWC) were calculated as follows:

$$LWC=\left(FW_{L}-DW_{L}\right)/FW_{L}\times 100\%$$ (1)

$$PWC=\left(FW_{P}-DW_{P}\right)/FW_{P}\times 100\%$$ (2)

Equivalent water thickness (EWT) is defined as the hypothetical thickness of a single layer of water over the whole leaf area (A):

$$EWT=\left(FW_{L}-DW_{L}\right)/A$$ (3)

At the canopy level, canopy water content (CWC) can be obtained by scaling the EWT with leaf area index (LAI):

CWC=EWT×LAI (4)

### Spectral indices

Random combinations of wavelengths in the range of 400–2400 nm were used to formulate different NDVI, DVI, and RVI in this study.

$$NDVI=\left(R_{i}-R_{j}\right)/\left(R_{i}+R_{j}\right)$$ (5)

$$DVI=R_{i}-R_{j}$$ (6)

$$RVI=R_{i}/R_{j}$$ (7)

Where *R*_*i*_ and *R*_*j*_ represent the reflectance of *i* and *j* nm.

Likewise, random combinations of the derivative reflectance of in the range of 400–2400 nm in the spectral indices,

$$FDNDVI=\left(FDR_{i}-FDR_{j}\right)/\left(FDR_{i}+FDR_{j}\right)$$ (8)

$$FDDVI=FDR_{i}-FDR_{j}$$ (9)

$$FDRVI=FDR_{i}/FDR_{j}$$ (10)

Where *FDR*_*i*_ and *FDR*_*j*_ represent the derivative reflectance of *i* and *j* nm.

The performance of the formulations from above were compared with nine published vegetation indices for the estimation of water status (Table 1).

**Table 1:** Published vegetation indices used in this study for water status estimation.

Vegetation indices	Name	Formula	Reference
WI	Water index	*R*_900_/*R*_970_	[8, 9]
WBI	Water band index	*R*_970_/*R*_900_	[8]
NDWI	Normalized different water index	(*R*_860_ − *R*_1240_)/ (*R*_860_ + *R*_1240_)	[20]
MSI	Moisture stress index	*R*_1600_/*R*_820_	[19]
NDII	Normalized different infrared index	(*R*_850_ − *R*_1650_)/(*R*_850_ + *R*_1650_)	[28]
WBI/NDVI		[*R*_970_/*R*_900_]/[(*R*_800_ − *R*_680_)/(*R*_800_ + *R*_680_)]	[23, 29]
PRI	Photochemical Reflectance Index	(*R*_531_ − *R*_570_)/(*R*_531_ + *R*_570_)	[30]
Red-edge NDVI	Red-edge normalized difference vegetation index	(*R*_750_ − *R*_705_)/(*R*_750_ + *R*_705_)	[22, 31]
OSAVI	Optimized soil-adjusted vegetation index	(1 + 0.16) × (*R*_800_ − *R*_670_)/(*R*_800_ + *R*_670_ + 0.16)	[25]

### Calibration and validation method

The prediction models were evaluated based on *R*^2^, the root mean square error (RMSE), and residual prediction difference (RPD). Accordingly, three crops of models were defined, adequate (RPD>2), acceptable (1.4<RPD<2), and inadequate (RPD<1.4) [32].

## Results

### The response of canopy spectrum to plant water status

**Fig 1:**
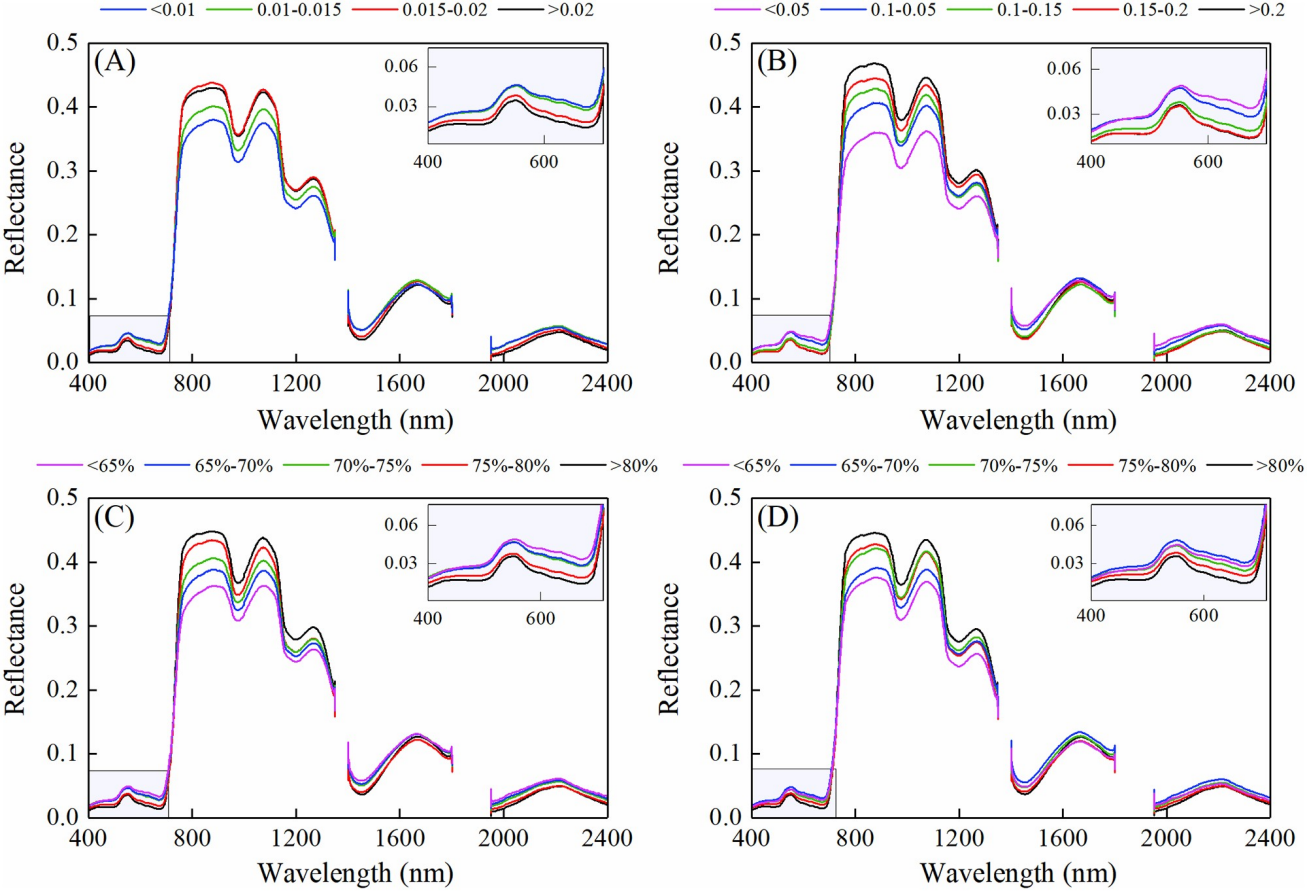
Canopy spectral reflectance (raw data) at various plant water status in winter wheat. Different colors indicate the mean spectrum of various gradients of water metrics. (A) equivalent water thickness (EWT), (B) canopy water content (CWC), (C)leaf water content (LWC) and (D) plant water content (PWC).

The raw reflectance spectral curves showed a similar trend (Fig 1). There was a positive relationship between reflectance and water status in the near-infrared wavelength and a negative relationship in the visible wavelength. As the water content decreased, reflectance of the red (approximately 680 nm) increased and that in the green (approximately 550 nm) became less obvious.

**Fig 2:**
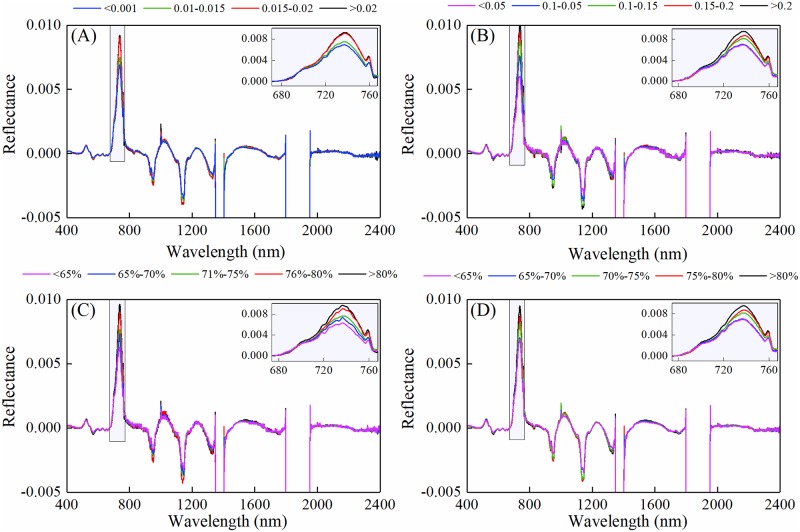
First derivative reflectance at various plant water status in winter wheat. Different color represents gradients of water metrics. (A) equivalent water thickness (EWT), (B) canopy water content (CWC), (C)leaf water content (LWC) and (D) plant water content (PWC).

Fig 2 shows the first derivative reflectance of different water metrics at various gradients. Spectral curves were differently affected by the chlorophyll and water absorption. The spectrum in 670–760 nm, was distinguished with a positive response to water metrics. These responses indicated that raw reflectance and the first derivative reflectance were sensitive to the change of crop water status in different spectral ranges.

### Relationship between canopy reflectance and plant water status

**Fig 3:**
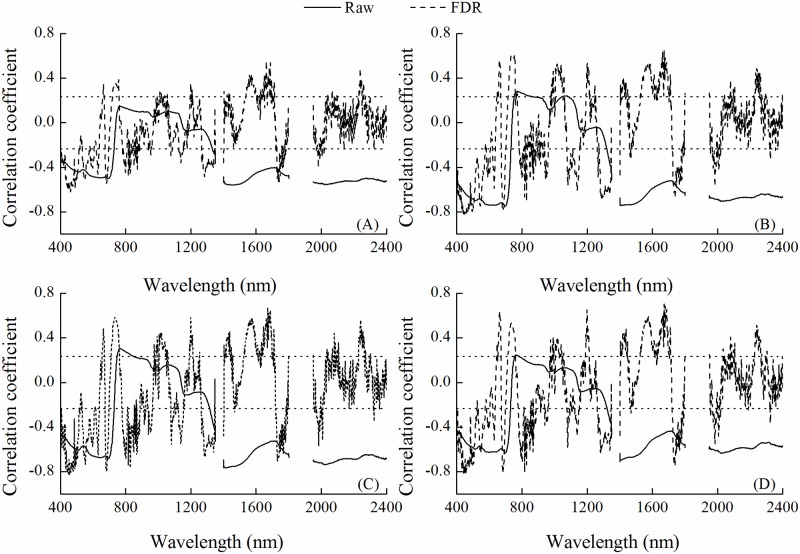
Correlation between water metrics and raw reflectance and between water metrics and the first derivative reflectance in winter wheat (n = 120). (A) equivalent water thickness (EWT), (B) canopy water content (CWC), (C) leaf water content (LWC) and (D) plant water content (PWC). The horizontal dotted lines represent the correlation coefficient threshold values at the 0.01 probability level.

Correlation coefficients between water metrics and each band were shown in Fig 3. And the correlation coefficients curves for different water metrics were in the same pattern. With exception in the range of 730–1315 nm, the raw reflectance showed significant negative correlations with water metrics. In Fig 3B and 3C, the positive correlation between water metrics (CWC and LWC) and spectrum was significant at the 0.01 probability level at 760 nm. The first derivative reflectance improves the correlation in the visible and near-infrared regions over the raw spectrum. The four water metrics had significant correlations with the raw reflectance and the first derivative reflectance.

### Relationships between plant water status and spectral indices

#### Relationships between plant water status and spectral indices using two raw bands

**Fig 4:**
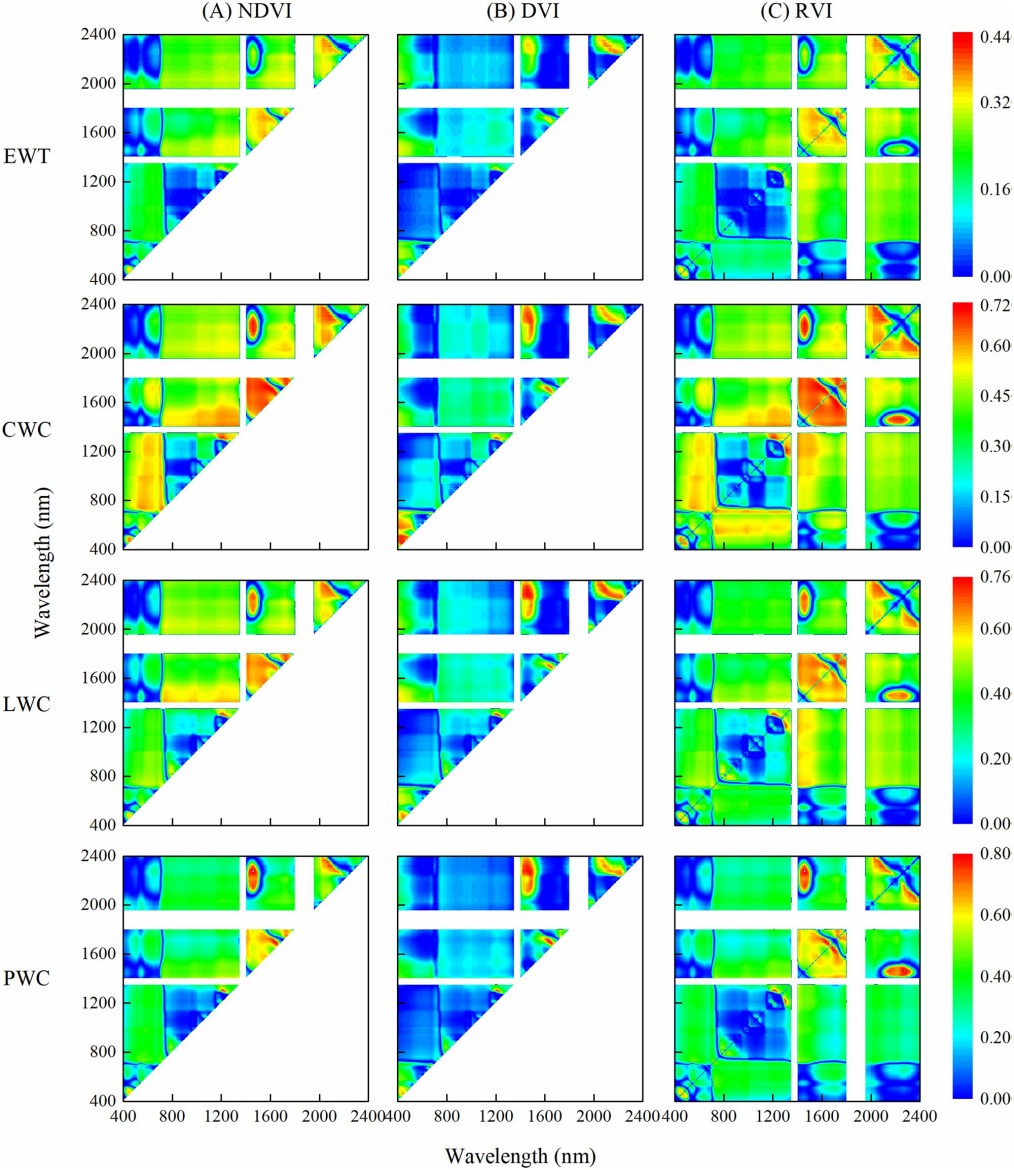
Coefficient of determination (R^2^) between plant water status and spectral indices using two bands of the raw reflectance in winter wheat.

The Fig 4 presented the *R*^2^ of linear regressions between water metrics and spectral indices calculated with random combination of two raw reflectance. The order of relationship between water metrics and optimal spectral index was PWC > LWC > CWC > EWT. The *R*^2^ values for different spectral indices of EWT were all below 0.44, while the *R*^2^ values for CWC, PWC and LWC were all above 0.73. For different water metrics, the patterns of contour maps of the same spectral index were almost same. Spectral indices NDVI and RVI showed similar contour map patterns. The optimal band combination indices for CWC and LWC were RVI (1605, 1712) and DVI (1301, 1213) with *R*^2^ of 0.74 and 0.76, respectively. Besides, NDVI and RVI had the same optimal combination of 2273 nm and 1460 nm with the R^2^ of 0.81 in predicting the PWC.

#### Relationships between plant water status and spectral indices using two bands with the first derivative

**Fig 5:**
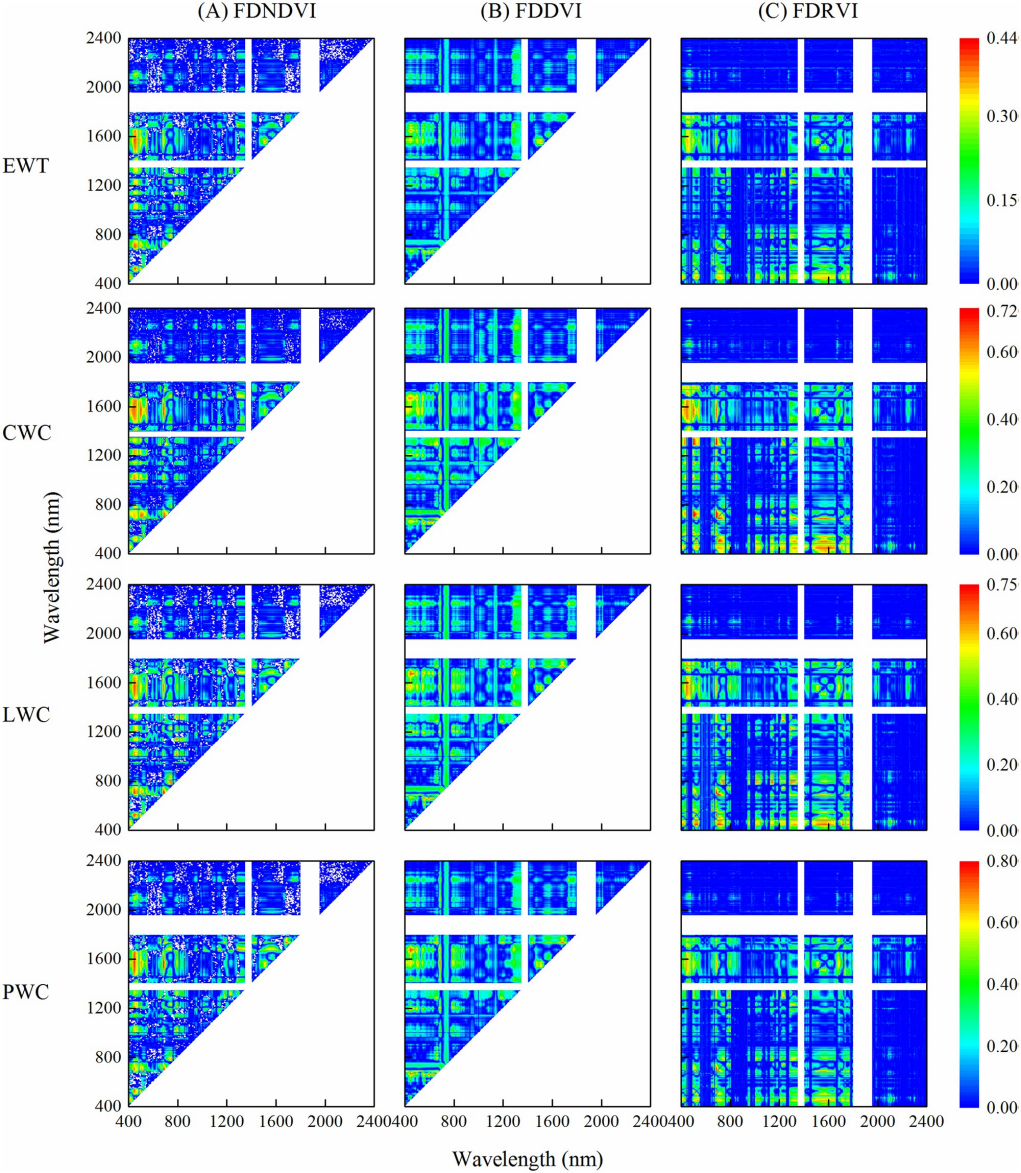
Coefficient of determination (R^2^) between plant water status and spectral indices using two bands of the first derivative reflectance in winter wheat.

R^2^ of linear regressions between different water metrics and spectral indices calculated with two derivative reflectance were shown in Fig 5. Compared with ordinary spectral indices, the orders of different water metrics were the same, while the combination ranges of derivative spectral indices which had good relationships with plant water status were different. The relationships between water metrics and derivative spectral indices (400–800 nm and 400–1800 nm) were closer than the ordinary spectral index. However, the best R^2^ of EWT was still lower than other water metrics, only 0.44. Optimal combination derivative spectral indices to predict CWC, LWC and PWC were FDRVI (713, 688), FDRVI (687, 531) and FDNDVI (688, 533), respectively.

### Calibration and validation of the quantitative model of plant water status based on new combination spectral index

The data from 2015–2016 and 2016–2017 were used for model calibration and validation, respectively. In order to select the optimal spectral indices for predicting the plant water status, the linear and nonlinear models of each water metric based on the new combination spectral index were established. The performances of quantitative models were compared (Table 2).

**Table 2:** Quantitative models of water metrics (y) to select spectral index (x) in winter wheat.

Water metrics	Spectral index	Formulation	Calibration			Validation		
			R^2^_C_	RMSE_C_	RPD_C_	R^2^_V_	RMSE_V_	RPD_V_
EWT	NDVI (2348, 2065)	y = 0.0455x + 0.0128	0.41	0.002	1.31	0.44	0.004	0.91
	FDNDVI (1560, 1505)	y = 0.0137e^1.9805x^	0.45	0.002	1.32	0.38	0.004	0.90
	DVI (2247, 2192)	y = -0.9937x + 0.0156	0.43	0.002	1.33	0.53	0.004	0.89
	FDDVI (1663, 489)	y = 23.443x + 0.0122	0.44	0.002	1.34	0.49	0.004	0.89
	RVI (2348, 2065)	y = -0.0226x + 0.0354	0.41	0.002	1.31	0.47	0.004	0.90
	FDRVI (1284, 1220)	y = 0.0086e^-0.433x^	0.45	0.002	1.31	0.54	0.003	1.07
CWC	NDVI (1712, 1605)	y = 4.2803x − 0.0158	0.73	0.027	1.94	0.71	0.029	1.79
	FDNDVI (1586, 687)	y = 0.2964x + 0.1651	0.74	0.026	1.98	0.70	0.031	1.69
	DVI (458, 445)	y = -76.353x + 0.1916	0.71	0.028	1.86	0.76	0.043	1.22
	FDDVI (663, 602)	y = 710.05x + 0.1733	0.71	0.028	1.85	0.63	0.039	1.33
	RVI (1605, 1712)	y = -11.927x^2^ + 20.155x − 8.2849	0.74	0.026	1.98	0.72	0.028	1.87
	FDRVI (713, 688)	y = 0.0355x − 0.0716	0.76	0.026	2.04	0.74	0.040	1.29
LWC	NDVI (1301, 1218)	y = -925.82x + 76.969	0.74	2.298	1.99	0.84	2.888	2.15
	FDNDVI (1015, 790)	y = 30.098x + 63.749	0.76	2.215	2.06	0.77	3.732	1.66
	DVI (1299, 1218)	y = -141188x^2^ + 1087.4x + 79.729	0.79	2.104	2.17	0.85	2.991	2.07
	FDDVI (669, 553)	y = 54751x + 77.873	0.74	2.324	1.96	0.72	3.857	1.61
	RVI (1301, 1218)	y = -460.59x + 537.57	0.75	2.295	1.99	0.84	2.882	2.15
	FDRVI (687, 531)	y = -18.567x + 97.6	0.77	2.181	2.09	0.87	2.652	2.34
PWC	NDVI (2273, 1460)	y = 0.0002x − 0.0048	0.81	2.978	2.32	0.85	4.315	1.87
	FDNDVI (688, 533)	y = -84.658x + 89.688	0.81	2.987	2.32	0.84	4.230	1.91
	DVI (1691, 1644)	y = 5183.1x + 71.246	0.81	3.003	2.30	0.80	5.836	1.38
	FDDVI (688, 532)	y = 6*10^7^x^2^ − 75018x + 85.153	0.79	3.136	2.21	0.83	3.702	2.18
	RVI (2273, 1460)	y = 28.118x^2^–131.97x + 176.08	0.81	2.970	2.33	0.85	4.329	1.87
	FDRVI (688, 533)	y = 8.8609x^2^–53.97x + 133.87	0.81	2.973	2.33	0.84	4.177	1.93

**Fig 6:**
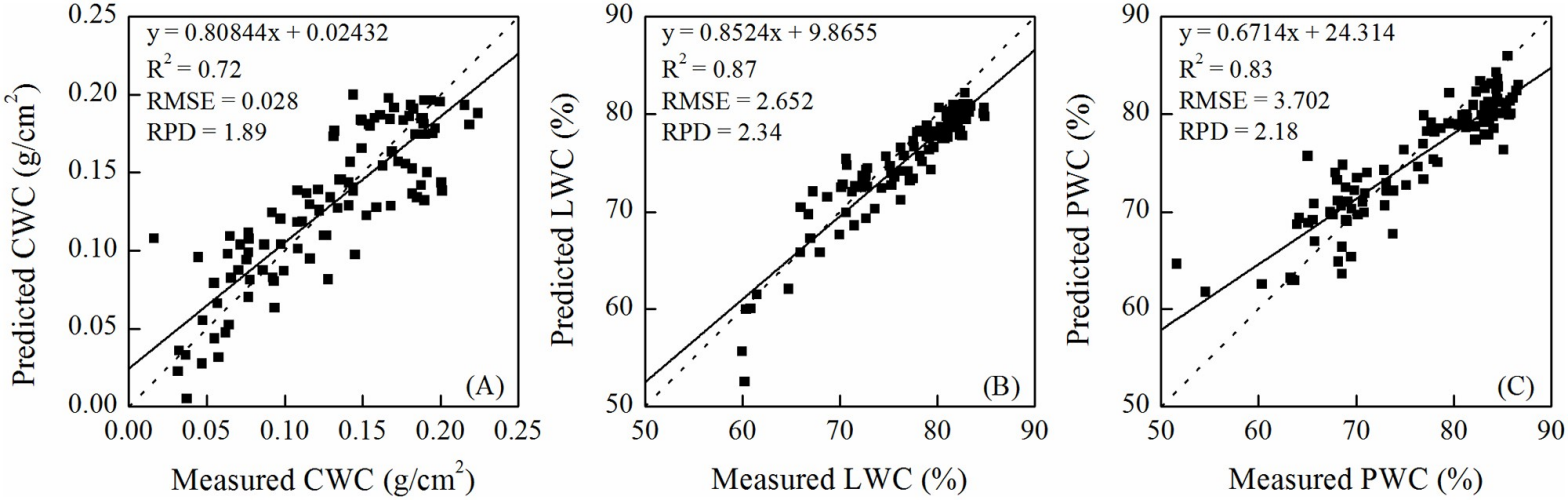
Scatter diagrams between the measured and estimated water metrics from the models with the optimal spectral index. (A) canopy water content (CWC), (B) leaf water content (LWC) and (C) plant water content (PWC). The 1:1 line is marked with a dotted line.

The quantitative relationships between the water metrics and the optimal band combination spectral indices were mostly linear models (Table 2). The predicting models for EWT showed low R^2^ (R^2^<0.45). The quantitative models for CWC were acceptable (R^2^>0.7 and 1.4<RPD<2.0). The best spectral index for CWC was RVI (1605, 1712). Models using the raw spectral indices were good in predicting LWC, while the validation of models based on derivative spectral indices were acceptable. However, the linear model using FDRVI (687, 531) was the optimal model of LWC having the highest R^2^, RPD and lowest RMSE in model calibration and validation (R^2^_C_ = 0.77, RMSE_C_ = 2.181, RPD_C_ = 2.09, R^2^_V_ = 0.87, RMSE_V_ = 2.652, RPD_V_ = 2.34) (Fig 6B). In addition, the performances of model calibration for predicting the PWC were also well. But, the performances of model validation were less (1.4<RPD<2.0), except for FDDVI (688, 532). Therefore, the nonlinear model using FDDVI (688, 532) was the optimal model of PWC having the higher R^2^, RPD and lowest RMSE in model calibration and validation (R^2^_C_ = 0.79, RMSE_C_ = 3.136, RPD_C_ = 2.21, R^2^_V_ = 0.83, RMSE_V_ = 3.702, RPD_V_ = 2.18) (Fig 6C).

### Performance of published vegetation indices

The correlation between various water metrics and published vegetation indices are listed in Table 3. Significant correlation was observed between published vegetation indices and CWC, LWC and PWC. And the correlation between CWC and vegetation indices were better than other water metrics. Except for WI, the relationships between vegetation indices and EWT were significant different at 0.05 level. The Red-edge NDVI had the highest correlation coefficients with water metrics. Then, vegetation indices with the first two correlation coefficients were selected to establish the predicting models (Table 4). Based on the RPD, the calibration models using published vegetation indices were not acceptable in this study. However, based on the new combination vegetation index (Table 2), the predicting models had the higher R^2^ and RPD and lower RMSE.

**Table 3:** Correlation coefficients between vegetation indices and water metrics (n = 120).

Vegetation indices	EWT	CWC	LWC	PWC
WI	0.178	0.423**	0.390**	0.276**
WBI	-0.183*	-0.428**	-0.400**	-0.281**
NDWI	0.217*	0.392**	0.438**	0.312**
MSI	-0.457**	-0.646**	-0.673**	-0.554**
NDII	0.426**	0.623**	0.635**	0.526**
WBI/NDVI	-0.515**	-0.679**	-0.679**	-0.601**
PRI	-0.462**	-0.694**	-0.655**	-0.569**
Red-edge NDVI	0.496**	0.776**	0.728**	0.670**
OSAVI	0.423**	0.662**	0.633**	0.550**

* and

** represent significant differences at 0.05 and 0.01 level, respectively.

**Table 4:** The performance of the calibration models based on the published vegetation indices.

Water metrics	Vegetation index	Model	R^2^	RMSE	RPD
EWT	Red-edge NDVI	y = 0.0169x^0.5928^	0.29	0.002	1.16
	WBI/NDVI	y = 0.033e^-0.69x^	0.30	0.002	1.18
CWC	Red-edge NDVI	y = 0.0039e^5.3849x^	0.68	0.033	1.56
	PRI	y = 0.1321e^-19.65x^	0.51	0.039	1.35
LWC	Red-edge NDVI	y = 87.149x^0.3034^	0.54	3.111	1.47
	WBI/NDVI	y = 88.51x^-0.5^	0.49	3.286	1.39
PWC	Red-edge NDVI	y = 45.229e^0.7759x^	0.46	5.105	1.35
	WBI/NDVI	y = 50.933x^2^–189.75x + 238.14	0.43	5.201	1.33

## Discussion

Canopy reflectance contains complex information. Both raw and derivative spectral reflectance had good correlations with various water metrics of winter wheat. With the decreasing values of water metrics, the raw reflectance in the near-infrared region and derivative reflectance in the red region decreased while the raw reflectance in the visible region increased (Fig 1), which is in agreement with previously published results [6, 33]. The increasing of near-infrared reflectance may be related to the geometrical features of leaf and canopy and intercellular scattering within the leaves [34]. The observed changes of water metrics in derivative reflectance in red region was due to the sensitivity of red edge parameters to water stress (Fig 2) [33].

Plant water status could be described with various physiological parameters [35]. All water metrics used in this study could be predicted by the raw and derivative reflectance. The effects of plant water status include plant growth, yield, pigment content, and photosynthetic activity [36], which would induce the changes of canopy reflectance. CWC, LWC and PWC had better relationships with raw and derivative spectrum than EWT (Fig 3). LWC and PWC, the correlation with spectrum and the performance of predicting model were similar. However, the LWC model had higher accuracy in model validation. Furthermore, the optimal spectral indices had a linear relationship with LWC, while a quadratic polynomial relationship with PWC. Because linear relationship has the advantage of the absence of spectral saturation effect [37], LWC was more suitable in the plant water status estimation by remote sensing.

Vegetation indices with adjustment of the influence of external factors (e.g. solar irradiance and soil background) improved the prediction accuracy of water status [18, 38]. In this study, random combinations of raw and derivative reflectance at 400–2400 nm were used to calculate new spectral indices. For the raw reflectance, spectral indices based on the near-infrared and short-infrared showed good performance in model calibration and validation [39–41]. However, derivative reflectance in visible and near-infrared regions were better suited for predicting plant water status in this study. Despite the noise interference, some researchers believed that derivative process could reduce the effects of radiation and soil background reflection on vegetation and approve the estimating accuracy [26, 42, 43].

## Conclusion

This study demonstrated the possibility of accurately predicting the plant water status by canopy reflectance. The canopy reflectance of winter wheat had good correlation with the water status of winter wheat. CWC, LWC and PWC could be adequately predicted by the spectral indices formulated in this study. Among four water metrics, LWC was the best water metric in assessing the plant water status. Meanwhile, having a linear relationship (y = -18.567x + 97.6) with LWC, FDRVI (687, 531) was the optimal spectral index with R^2^, RMSE and RPD of 0.77, 2.181 and 2.09; R^2^, RMSE and RPD of 0.87, 2.652 and 2.34 for calibration and validation, respectively.

## Supporting information

S1 Data Data of Figs 1–3 and 6. (XLSX)

S2 Data Data of Fig 4 EWT and CWC. (XLSX)

S3 Data Data of Fig 4 LWC and PWC. (XLSX)

S4 Data Data of Fig 5 EWT and CWC. (XLSX)

S5 Data Data of Fig 5 LWC and PWC. (XLSX)

S6 Data Data of Tables 2–4. (XLSX)
